# First-principles determination of intergranular atomic arrangements and magnetic properties in rare-earth permanent magnets

**DOI:** 10.1080/14686996.2021.1877092

**Published:** 2021-02-12

**Authors:** Yoshihiro Gohda

**Affiliations:** Department of Materials Science and Engineering, Tokyo Institute of Technology, Yokohama, Japan

**Keywords:** First-principles calculations, electron theory, ferromagnetism, permanent magnets, Curie temperature, exchange coupling, microstructure, interfaces, 403 Electronic structure calculations, 203 Magnetics / Spintronics / Superconductors, 401 1st, principles methods

## Abstract

Development of high-performance permanent magnets relies on both the main-phase compound with superior intrinsic magnetic properties and the microstructure effect for the prevention of magnetization reversal. In this article, the microstructure effect is discussed by focusing on the interface between the main phase and an intergranular phase and on the intergranular phase itself. First, surfaces of main-phase grains are considered, where a general trend in the surface termination and its origin are discussed. Next, microstructure interfaces in SmFe_12_-based magnets are discussed, where magnetic decoupling between SmFe_12_ grains is found for the SmCu subphase. Finally, general insights into finite-temperature magnetism are discussed with emphasis on the feedback effect from magnetism-dependent phonons on magnetism, which is followed by explanations on atomic arrangements and magnetism of intergranular phases in Nd-Fe-B magnets. Both amorphous and candidate crystalline structures of Nd-Fe alloys are considered. The addition of Cu and Ga to Nd-Fe alloys is demonstrated to be effective in decreasing the Curie temperature of the intergranular phase.

## Graphical Abstract

**Figure:**
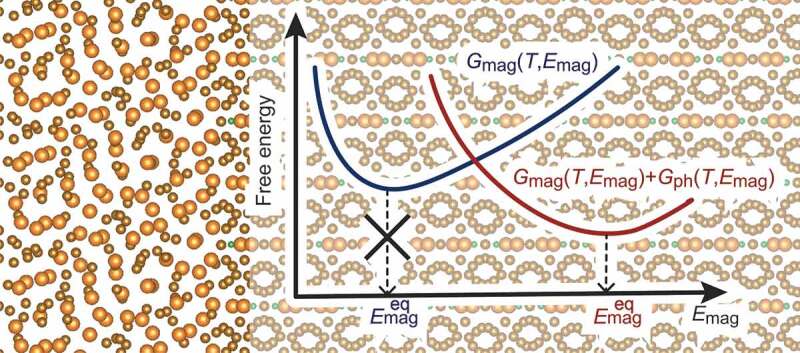

## 1. Introduction

High performance of rare-earth permanent magnets such as Nd-Fe-B magnets [1–3] is attributed to a magnetic compound with superior intrinsic magnetic properties [4,5] and to microstructures suppressing magnetization reversal [6–15]. In this article, the latter point is focused on. As a primary design principle of microstructures, it is well accepted as preferable to suppress the magnetic interaction between main-phase grains by non-magnetic subphases. Thus, it is of importance to determine the atomic arrangements and magnetism of intergranular subphases. In addition, the interplay between the intergranular subphase and the main phase depends on the atomic configuration at the interface between them. The propagation of magnetic domain-walls into a main-phase grain occurs through its surface. Moreover, due to the microstructure formation by the solidification of intergranular subphases, surface structures strongly influence atomic arrangements at microstructure interfaces. Considering this situation, surfaces of main-phase grains as well as interfaces between the main-phase and an intergranular subphase are of interest for microscopic understanding of magnetization reversal. Of course, structural and magnetic properties of suitable intergranular subphases must be first identified before proceeding to surfaces and interfaces.

From the viewpoint of electron theory from first principles, standard approximations such as the generalized gradient approximation (GGA) for the exchange-correlation functional in density functional theory (DFT) have been widely used for the identification of crystal structures and magnetic properties of magnetic materials. The magnetic ground states of bcc Fe and Fe-based alloys can be recognized as itinerant with rather wide d bands, which has been confirmed by photoemission spectra. In contrast, magnetic thermal excitation in Fe can be interpreted mainly as transverse fluctuation of localized spin densities at atomic sites. It seems that this fluctuation of the atomic spin direction is caused by strong Hund’s couplings coming from nuclear-electron attraction, not from electron-electron repulsion [16]. Thus, finite-temperature magnetism is often treated by effective spin-lattice models such as the Heisenberg model, with parameters derived from first principles [17,18] for Fe-based alloys as well as permanent-magnet compounds [19–23]. As for rare-earth elements, strongly localized and, thus, strongly correlated 4f electrons are beyond the applicability of standard exchange-correlation functionals in first-principles calculations, where these functionals incorporate weak correlation in itinerant electrons. A widely used approximation is the open-core approximation, where the 4f states remain unchanged as atomic states. Another possibility is to use a scheme taking strong electron correlation into account: permanent-magnet materials have been calculated by DFT combined with dynamical mean-field theory (DFT+DMFT) [24–26], where on-site correlation is added onto the DFT+U method dynamically. It is also straightforward to include magnetic thermal excitation by the DFT+DMFT method. Furthermore, spin-fluctuation theory [27] has been combined with first-principles calculations [28]. Nevertheless, quantitative descriptions of finite-temperature magnetism for realistic magnetic compounds in permanent magnets are far from being satisfactory as discussed below. Moreover, past first-principles studies mostly focused on the main phases of permanent magnets overlooking microstructure effects.

On the basis of electron theory, this paper discusses microstructural properties related to intergranular phases in permanent magnets as well as finite-temperature magnetism. Section 2 is devoted to general trends in surface structures of magnetic compounds including rare-earth elements and transition metals, as the main phase of permanent magnets. SmFe${\;}_{12}$ is chosen as an example. In Section 3, magnetic coupling among main-phase grains separated by an intergranular phase is discussed by heterostructure models for SmFe${\;}_{12}$-based magnets. Large-scale computations for Nd-Fe-B magnets are also discussed. In Section 4, recent attempts for better descriptions of finite-temperature magnetism are discussed first focusing on the effect of magnetism-dependent phonons on magnetism. Then, for Nd-Fe-B magnets, atomic arrangements and magnetism of intergranular subphases depending on the alloy composition are considered. Insights are given for the effect of the addition of the third element on microscopic exchange couplings and the Curie temperature of the intergranular phase. Section 5 summarizes the paper.

## 2. Surfaces of main-phase grains

Surfaces of main-phase grains are of importance to prevent the propagation of magnetic domain walls, because the inner part of main-phase grains cannot pin the domain-wall motion. Atomic configurations at surfaces of main-phase grains should remain unreconstructed by the sintering process in the production of permanent magnets, because of the subphase solidification on main-phase surfaces. First-principles calculations based on DFT are suitable to obtain atomistic information such as the most stable surfaces of main-phase grains. Computational results for SmFe${\;}_{12}$ by using the OpenMX code [29] are shown as an example of a general trend for the stable surfaces. The GGA is employed for the exchange-correlation functional. Open-core pseudopotentials for rare-earth elements are used in the treatment of 4f states. To impose the periodic boundary condition for the atomic structure and the electron density, the periodic-slab model is employed, where the surface slabs are repeatedly configured in vacuo with the separation of more than 10 Å. The lateral lattice constants and atomic positions are optimized by calculating the stress tensor and forces on atoms, respectively.

**Figure 1.**
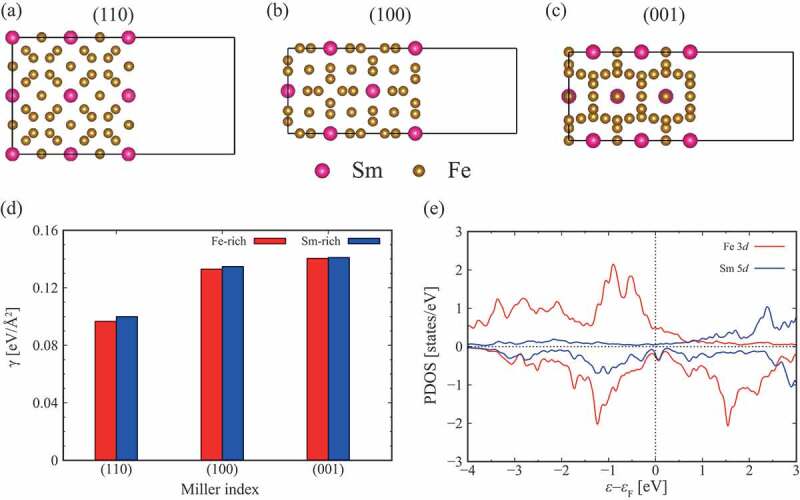
(a-c) Surface structures and (d) surface-energy densities γ of SmFe${\;}_{12}$. (e) Projected density of states (DOS) for 5d states of the Sm atoms and 3d states of the Fe atoms in the SmFe${\;}_{12}$ bulk. The upper (lower) panel denotes the projected DOS of the majority (minority) spin, respectively. The single-electron energy is defined relative to the Fermi energy $\varepsilon_{F}$. The figure is adapted from [30]. Copyright (2020) The Authors

Figures 1(a-c), show the most stable surface atomic configurations for each surface index by considering all possible terminations. This result is independent of the choice of the chemical potentials, the possible range of which is determined by the following formulae

$$E_{SmFe_{12}}=\mu_{Sm}+12\mu_{Fe}$$

$$\mu_{Sm}\leq E_{\alpha -Sm}$$

$$\mu_{Fe}\leq E_{bcc-Fe},$$

where $E_{SmFe}$_12_ is the total energy of the SmFe${\;}_{12}$ bulk single crystal per formula unit, $E_{\alpha -Sm}$ is that of α-Sm, and $E_{bcc-Fe}$ is that of bcc Fe, as well as $\mu_{Sm}$ and $\mu_{Fe}$ are the chemical potentials of Sm and Fe, respectively. To examine the stability among surface indices, the surface-energy density γ of SmFe${\;}_{12}$ surfaces is evaluated by

$$\gamma =\frac{E_{slab}-\left(N_{Sm}\mu_{Sm}+N_{Fe}\mu_{Fe}\right)}{2A},$$

where $E_{slab}$ is the total energy of the surface slab, $N_{i}$ is the number of atoms for the element i within the slab, and A is the surface area. Figure 1(d) shows γ for the three low-index surfaces with the Fe-rich and Sm-rich conditions. The Fe-rich condition corresponds to $\mu_{Fe}=E_{bcc-Fe}$ meaning that SmFe${\;}_{12}$ equilibrates with bcc Fe. As the opposite limit, the Sm-rich condition, $\mu_{Sm}=E_{\alpha -Sm}$, means that SmFe${\;}_{12}$ equilibrates with α-Sm. Independent of the choice of the chemical potentials, it is evident that the (110) surface is the most stable.

Furthermore, it is remarkable to see that the most stable surfaces are always terminated by planes with Sm atoms irrespective of surface indices [30]. This surface stabilization by rare-earth atoms comes from weaker chemical bindings for rare-earth 5d states than for Fe 3d states. Figure 1(e) shows the density of states (DOS) projected onto Sm 5d states and that onto Fe 3d states for the SmFe${\;}_{12}$ bulk single crystal. From the height of the DOS, it is clear that the binding energy of 5d states of Sm atoms is lower than that of Fe 3d electrons. Indeed, 1.4 electrons in the Sm 5d states are significantly fewer than 6.7 electrons in the Fe 3d states, which were identified for the SmFe${\;}_{12}$ bulk by the Mulliken-population analysis. It is remarkable that this trend of the energy-loss minimization in the 3d-bands seems to be universal, because Nd${\;}_{2}$Fe${\;}_{14}$B surfaces have terminations with Nd atoms [30].

## 3. Interfaces between the main phase and an intergranular phase

**Figure 2.**
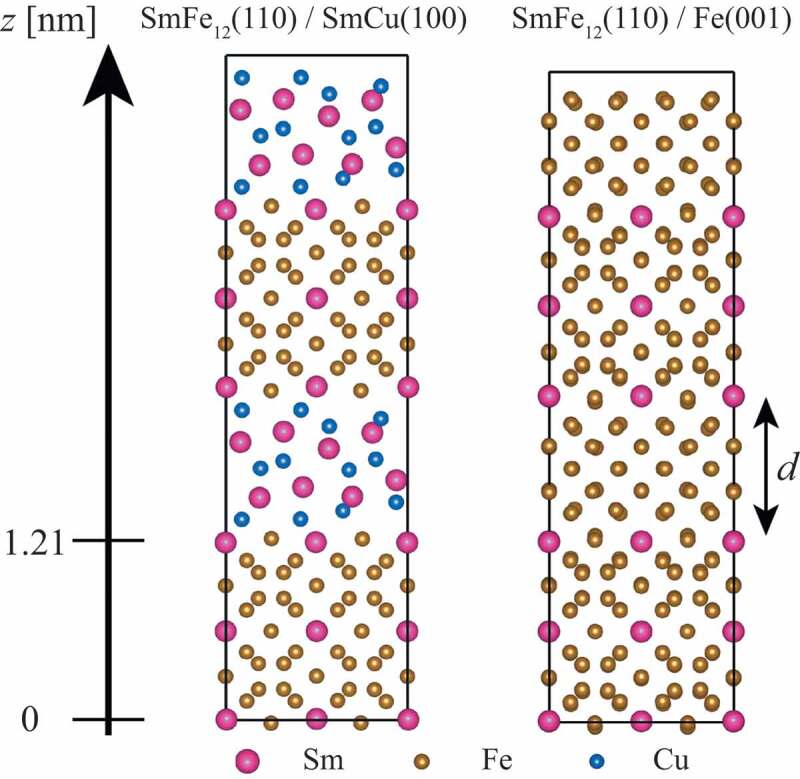
Optimized interface structures. The position with z=0 and 1.21 nm represents the first interface layer of SmFe${\;}_{12}$. d is the distance between SmFe${\;}_{12}$ grains defined as that between the first interface layers. The figure is adapted from [30]. Copyright (2020) The Authors

Nd-Fe-B permanent magnets have advantages in microstructures, where Nd-rich alloys with relatively low melting temperatures can be utilized. In contrast, the situation in SmFe${\;}_{12}$-based magnets [31,32] is not the case: such a suitable liquid phase possible to coexist with SmFe${\;}_{12}$ is yet to be established [33]. Depending on the composition, the primary intergranular subphase of SmFe${\;}_{12}$-based magnets becomes unfavorably bcc Fe [34]. Another possibility for the intergranular subphase is SmCu, as we examine the Gibbs free energy for Sm-Fe-Cu ternary systems combining the CALPHAD approach with first-principles calculations. At high temperatures, liquid SmCu can coexist with SmFe${\;}_{12}$ in the Sm-Fe-Cu phase diagram, assuming the stabilization of SmFe${\;}_{12}$ by, e.g., the addition of Ti. Here, the effects of intergranular phases are compared for SmFe${\;}_{12}(110)/$SmCu(100) and SmFe${\;}_{12}(110)/$Fe(001) interfaces. First, the atomic arrangements are determined by a typical heterostructure model consisting of a main-phase slab and a subphase slab within a supercell, i.e., a large box with the periodic boundary condition to model an interface. Then, magnetic coupling among main-phase grains is examined by doubling the supercell in the interface-normal direction as depicted in Figure 2. These supercells include nearly 550 atoms. By changing relative spin configurations of the two main-phase grains in the doubled supercell, exchange interaction among main-phase grains is evaluated from the total energy. This effective exchange interaction is compared for SmFe${\;}_{12}(110)/$SmCu(100) and SmFe${\;}_{12}(110)/$Fe(001) with almost the same subphase thickness: the distance between SmFe${\;}_{12}$ grains d is d=10.3 Å for SmFe${\;}_{12}$/SmCu and d=9.5 Å for SmFe${\;}_{12}$/Fe. As a result, it is found that the exchange interaction between main-phase grains is 12 times smaller for SmFe${\;}_{12}$/SmCu than SmFe${\;}_{12}$/Fe [30]. This result makes it clear that grains of the main phase are magnetically decoupled by SmCu in contrast with Fe.

**Figure 3.**
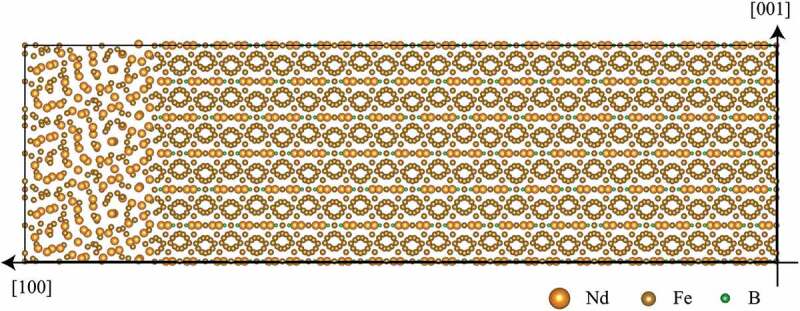
Interface supercell with 8463 atoms for the main phase and an intergranular phase in Nd-Fe-B magnets

The microscopic characterization of interfaces between the main phase and an intergranular phase is helpful in understanding the domain-wall motion, because the surfaces of a main-phase grain are only the last stage to prevent magnetization reversal of the grain. Since the atomic configuration is at the local minimum in potential-energy surfaces, the atomic structure optimization is indispensable for precise calculations, so that the electronic structure far from the interface reproduces that of bulks. At present, practical first-principles calculations of interfaces between the main phase and an intergranular phase are limited for clean ones with low Miller indices [12,30,35,36]. However, atomic arrangements of interfaces are diverse, because microstructure interfaces are far from thermodynamical equilibrium. Even though molecular dynamic simulations based on interatomic model potentials have been employed to construct models for microstructure interfaces [37–39], equivalent calculations from first principles are still very challenging in general. This situation enforces us to perform large-scale first-principles computation using supercomputers such as the K Computer and the supercomputer Fugaku. Figure 3 shows a supercell used for large-scale first-principles computation in the K computer with the OpenMX code [29]. In this calculation, 8463 MPI parallelizations together with 8 OpenMP parallelizations are employed enabling one self-consistent-field loop to optimize the electronic structure within 20 minutes. Such large-scale computations are useful for high-index interfaces with disorders as well as for low-index interfaces with a large lattice mismatch. Local magnetic properties such as the magnetic moment, the magnetocrystalline anisotropy, and the exchange coupling constant at a variety of interfaces can be utilized for atomistic spin-lattice models.

## 4. Finite-temperature magnetism for intergranular phases in Nd-Fe-B permanent magnets

For Nd-Fe-B permanent magnets, it has been long believed that intergranular phases are simply rich in Nd and paramagnetic. More detailed experimental insights into the composition and magnetism of intergranular phases have been obtained relatively recently [40–43]. In particular, the surface orientation of the main-phase grains is pointed out to play a crucial role in determining the composition and the atomic arrangements as well as magnetism of Nd-Fe alloys as intergranular phases [42]. For example, the intergranular phase contacting with non-(001) surfaces of the main phase has been reported as amorphous and ferromagnetic [40,44]. The Nd composition of the amorphous Nd-Fe phase is typically 30∼40at.%. In contrast, the intergranular phase becomes crystalline on the (001) surface of Nd${\;}_{2}$Fe${\;}_{14}$B. In this case, the intergranular phase is paramagnetic at room temperature with the Nd composition of 60 to 70at.% [42]. Nanobeam-electron-diffraction and the Fourier-transformed scanning-transmission-electron-microscopy patterns indicate that the crystal structure of the Nd-Fe intergranular phase is the fcc type [43]. However, detailed information on the crystal structure has not been available experimentally.

Furthermore, the present-day theoretical description of finite-temperature magnetism is insufficient in general. Thus, problems and recent progress for this issue are discussed first, before proceeding to the magnetic interaction in Nd-Fe intergranular phases.

### 4.1. Finite-temperature magnetism

**Figure 4.**
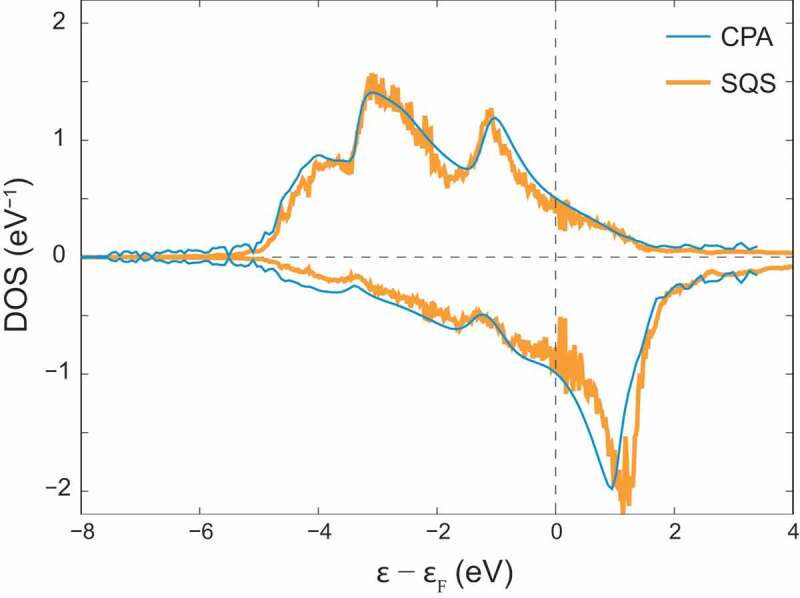
Local DOS at an up-spin site in bcc Fe for the LMD state modeled by the SQS method and by the CPA. Positive (negative) DOS is for the up (down) spins, respectively. In the SQS method, 3×3×3 supercell having 54 atoms are used, while the primitive cell with a single atom is used with the CPA

Ultimate theory for finite-temperature magnetism is not yet available. As one of the most practical treatments, magnetic interactions among atoms can be simplified as spin–spin interaction with effective exchange couplings. Assuming that magnetic interactions can be mapped onto effective spin-lattice models with the exchange-coupling constant $J_{ij}$, the spin dependence of the total energy can be reduced to the magnetic energy $E_{mag}$ described by the Heisenberg model:

$$E_{mag}=-\sum_{i}\sum_{j\neq i}J_{ij}\langle {\hat{s}}_{i}\cdot {\hat{s}}_{j}\rangle ,$$

where ${\hat{s}}_{i}$ is the unit vector representing the spin direction of the site i. It should be noted, however, that the mapping of magnetic interaction on the Heisenberg model is not trivial for 5d electrons in rare-earth elements, because it assumes magnetic excitation of local magnetic moments with strong Hund’s couplings. As magnetic force theorem, Liechtenstein’s formula [18] is widely used to calculate $J_{ij}$ from first principles. As a reference state in calculating $J_{ij}$, the magnetic ground state is usually adopted. Another choice is the so-called local moment disorder (LMD) state that is also referred to as the disordered local moment (DLM) state [17]. The LMD/DLM state has randomly oriented magnetic moments at atomic sites corresponding to ideal Curie–Weiss paramagnetism in the high-temperature limit of the magnetic order, where fluctuations in the spin direction occur sufficiently slowly compared with other electronic degrees of freedom: electronic states are in their ground state for given spin configurations. Within collinear magnetism, the LMD state can be described as a two-component random alloy having up-spin sites and down-spin sites that can be efficiently modeled by the special quasirandom structure (SQS) method [45]. Alternatively, the randomness can be implicitly considered by the coherent potential approximation (CPA) avoiding large periodic supercells, where the Korringa-Kohn-Rostoker (KKR) method is often employed to calculate $J_{ij}$ from first principles, e.g., as implemented in the Akai-KKR code [46]. Figure 4 shows local DOS at an up-spin site in bcc Fe for the LMD state modeled by the SQS method and by the CPA. The agreement between the two methods looks satisfactory, which should be related to the fact that the CPA works relatively well for high alloy compositions.

One of the most crucial problems in the above scheme is that the most significant temperature effect, i.e., the influence of lattice vibrations is neglected. To incorporate lattice vibrations, local phonon disorder [47] and LMD has been treated at the same time in the framework of KKR-CPA [48]. The effect of phonons on the Curie temperature $T_{C}$ of bcc Fe has been studied from first principles by considering the dependence of $J_{ij}$ on phonons [49–51] or the dependence of the self-energy on phonons in spin-fluctuation theory [28]. However, the impact of phonons still remains controversial: even though considerable changes in $T_{C}$ by displacement-dependent $J_{ij}$ has been reported [51], other studies have reported that this effect on $T_{C}$ is sufficiently small [28,49,50]. In addition, since $J_{ij}$ is evaluated in a perturbative manner, it depends on a reference magnetic state, where the magnitude of $J_{ij}$ increases qualitatively as the magnetic disorder increases. On the contrary, the magnitude of displacement-dependent $J_{ij}$ decreases as the temperature increases. Summarizing these findings, it is fair to say that the temperature dependence of $J_{ij}$ is still unknown. Furthermore, in the above studies, the dependence of phonons on magnetism was ignored. We will see that it is important to consider the feedback effect on magnetism from phonons that are dependent on magnetic states. For this purpose, the Gibbs free energy at temperature T is minimized by variation in the magnetic energy $E_{mag}$, which is expressed for zero external fields as

$$G(T)=\underset{E_{mag}}{\min}\left[E_{mag}-TS_{mag}(E_{mag})+G_{ph}(T,E_{mag})\right].$$

It seems that the magnetization cannot be a good order parameter, because the paramagnetic state just above the Curie temperature $T_{C}$ and the LMD state have both zero magnetization with different microscopic states: the paramagnetic state just above $T_{C}$ has short-range order of magnetic moments. See Ref [52] for more details of the thermodynamic formulation with finite external fields. The magnetic entropy $S_{mag}(E_{mag})$ can be evaluated by Monte Carlo simulations incorporating quantum-spin effects [53]. The free energy of magnetism-dependent phonons $G_{ph}(T,E_{mag})$ is described with phonon frequencies as a function of $E_{mag}$:

$$\;\;\;\;\;\;\;\;\;\;\;\;\;\;\;\;\;\;\;\;\;\;\;\;\;\;\;\;\;\;\;\;\;\;\;\;\;\;\;\;\;\;\;\;\;\;\;\;\;\;\;\;\;\;\;\begin{array}{r} G_{ph}(T,E_{mag})=k_{B}T\frac{V}{{\left(2\pi \right)}^{3}}\text{\textbackslash{}break}\;\;\;\;\;\;\;\;\;\;\;\;\;\;\;\;\;\;\;\;\sum_{j}\int dq\log\\ \left[2\sinh\left(\frac{\hbar \omega_{qj}(E_{mag})}{2k_{B}T}\right)\right], \end{array}$$

where $k_{B}$ is the Boltzmann constant, V is the volume, ℏ is the reduced Planck constant, and $\omega_{qj}(E_{mag})$ is the phonon frequency of the j-th branch at the wave vector q as a function of $E_{mag}$. The phonon frequency is calculated by interatomic force constants $\Phi_{ij}(E_{mag})$ between sites i and j that are obtained using the linear interpolation of $\Phi_{ij}(E_{mag}^{FM})$ and $\Phi_{ij}(E_{mag}^{PM})$, where $E_{mag}^{FM}$ is $E_{mag}$ for the ground state in the ferromagnetic limit and $E_{mag}^{PM}$ is that for the LMD state in the paramagnetic limit [54]. Assuming that the timescale for phonons is always much longer than that for magnetic fluctuation, $\Phi_{ij}(E_{mag})$ retain the crystal symmetry irrespective of magnetic order [55]. Using force-displacement data sets obtained from first principles, $\Phi_{ij}(E_{mag})$ can be calculated by using, e.g., the ALAMODE code [56]. For the LMD state, many force-displacement data sets are necessary to avoid biased sampling.

**Figure 5.**
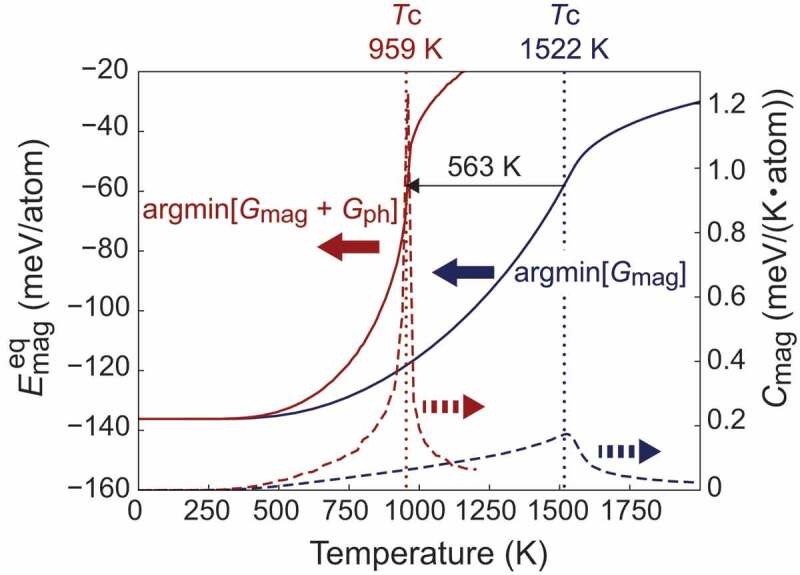
The equilibrium magnetic energy (solid lines) and the magnetic specific heat (dashed lines) of bcc Fe as a function of temperature. The LMD state is used as a reference state in determining $J_{ij}$. The orange lines represent the results obtained by the minimization of the total free energy, whereas the blue lines represent the results obtained by the minimization of the magnetic free energy. The figure is adapted from [52]. Copyright (2020) The Authors

For the prediction of $T_{C}$, singular magnetic fluctuation is identified in the magnetic specific heat

$$C_{mag}(T)=\frac{dE_{mag}^{eq}(T)}{dT},$$

where

$$E_{mag}^{eq}(T)=\underset{E_{mag}}{argmin}\left[G_{mag}(T,E_{mag})+G_{ph}(T,E_{mag})\right]$$

and $G_{mag}(T,E_{mag})=E_{mag}-TS_{mag}(E_{mag})$. It should be noted that the temperature T is independent of $E_{mag}$, and, thus, of the temperature used in Monte Carlo simulations to evaluate $E_{mag}$. Applying this formalism to bcc Fe [52], surprising reduction in $T_{C}$ by more than 500 K is obtained by considering magnetism-dependent phonons (Figure 5). This result indicates that interaction between magnetism and phonons is not negligible at all in general in determining $T_{C}$. Since the phonon effects stabilize the paramagnetic state, $T_{C}$ obtained only by the Heisenberg model must be much higher than experimental $T_{C}$, 1043 K in the case of bcc Fe, to be consistent with experiments. In this sense, the LMD state is more suitable than the ground state as a reference state to determine $J_{ij}$ due to the $T_{C}$ overestimation [17,51,52]. To understand the prominent stabilization in the paramagnetic state due to phonons, a concept called the exchange ligand field can be useful [57]. Developments of theoretical methods for finite-temperature magnetism (both the indirect effect of magnetism-dependent phonons on magnetism and the direct effect of atomic displacements and magnetic states on $J_{ij}$) and applications to magnetic compounds involved in permanent magnets should stimulate further progress providing feedbacks with each other.

### 4.2. Amorphous intergranular phase

Neodymium magnets have Nd-Fe amorphous alloys as the intergranular phase facing with non-(001) planes of the main phase, Nd${\;}_{2}$Fe${\;}_{14}$B [43]. For fcc and bcc structures, Nd-Fe substitutional random alloys were examined from first principles [44]. Indeed, first-principles studies of amorphous structures have been quite rare for permanent magnets, because past studies mostly focused on main phases that are crystalline [19–23,31,58–62]. Amorphous structures within a supercell can be obtained by first-principles molecular dynamics with the melt-quench approach [63]. Even though the distribution of interatomic distances is continuous in amorphous structures, it is possible to identify the nearest-neighbor pairs by utilizing Gabriel graphs [64,65]. The average of the interatomic distances for, e.g., the nearest-neighbor Nd-Fe pairs in amorphous Nd${\;}_{x}$Fe${\;}_{1-x}$ alloys is obtained as $\langle r_{Nd-Fe}\rangle =3.2$ Å that hardly depends on x within 0.2≤x≤0.8.

For qualitative discussions of finite-temperature magnetism, the computation of the Curie temperature can be extremely simple: only the spin Hamiltonians are used with neglecting the feedback effect from magnetism-dependent phonons discussed above, where $T_{C}$ is evaluated by the mean-field approximation instead of Monte Carlo simulations. Within the mean-field approximation, $T_{C}$ can be estimated as the maximum eigenvalue of the matrix, whose element is in general defined by

$$\frac{2}{3k_{B}}J_{ij}.$$

The dimension of the matrix is finite in practical computations due to the periodicity of supercells, where $J_{ij}$ values in the same magnetic sublattice are summed up as the zero-wave-vector Fourier component. In the case of amorphous structures, magnetic sublattices are simply defined by the lattice vectors of the supercell.

**Figure 6.**
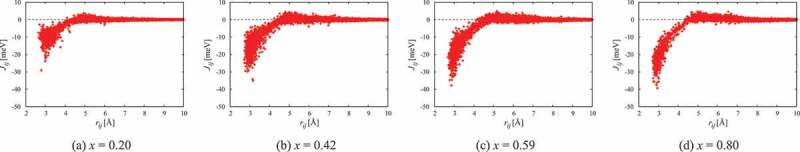
Exchange coupling constant $J_{ij}$ between Nd-Fe pairs as a function of the interatomic distance $r_{ij}$ for amorphous Nd${\;}_{x}$Fe${\;}_{1-x}$ alloys with (a) x=0.20, (b) x=0.42, (c) x=0.59, and (d) x=0.80

Other expressions of the Liechtenstein method [18] to calculate $J_{ij}$ have been given without using t-matrix in order to apply the method to first-principles schemes that do not directly use the concept of multiple scattering [66]. For amorphous Nd-Fe alloys, $J_{ij}$ has been evaluated by a formalism that accelerates the computation of $J_{ij}$ with non-orthogonal basis sets [67]. Figure 6 shows $J_{ij}$ between Nd-Fe pairs as a function of the interatomic distance $r_{ij}$ for amorphous Nd${\;}_{x}$Fe${\;}_{1-x}$ alloys, where $J_{ij}$ is derived from the magnetic ground state. In the magnetic ground state, aligned Fe spin moments couple antiparallel with aligned Nd spin moments. Considering 4f orbital magnetic moments, the coupling among total moments becomes ferromagnetic. Here, $J_{ij}$ is defined for spins, while only the sign of $J_{ij}$ changes for interactions between total magnetic moments. It is remarkable to see from the figure that the fluctuation of the nearest-neighbor Nd-Fe interatomic distance $r_{Nd-Fe}$ from the average strongly changes the values of $J_{ij}$ for nearest-neighbor Nd-Fe pairs, $J_{Nd-Fe}$. The magnitude of $J_{Nd-Fe}$ increases nearly exponentially as $r_{Nd-Fe}$ becomes shorter. This tendency is clearer as x becomes larger. As a result, the average of $J_{Nd-Fe}$, $\left\langle J_{Nd-Fe}\right\rangle$, depends strongly on x: $\langle J_{Nd-Fe}\rangle =-9$ meV for x=0.2, while $\langle J_{Nd-Fe}\rangle =-18$ meV for x=0.8 [68]. It should be noted that the large magnitude of the magnetic interaction between Nd and Fe is remarkable in comparison with Nd${\;}_{2}$Fe${\;}_{14}$B that has $J_{Nd-Fe}$ up to −5 meV [22]. The identical trend of stronger exchange interaction for large x is seen for $\left\langle J_{Fe-Fe}\right\rangle$. Consequently, $T_{C}$ is almost independent of x for x<0.5, even though the number of nearest-neighbor Fe-Fe pairs decreases with increasing x. However, $T_{C}$ decreases for x>0.5 as x becomes larger, because both of the numbers of nearest-neighbor Fe-Fe and Nd-Fe pairs decrease.

### 4.3. Crystalline intergranular phase

As a candidate for the crystalline intergranular phase in Nd-Fe-B permanent magnets, various crystal structures for Nd-Fe alloys are considered as fcc-type intergranular phases. For stoichiometric Nd${\;}_{2}$Fe, i.e., Nd${\;}_{0.67}$Fe${\;}_{0.33}$, first-principles calculations identify the fluorite structure as the most stable among tested crystal structures. Then, the variation in the composition as Nd${\;}_{x}$Fe${\;}_{1-x}$ is examined by the atom substitution as well as the vacancy formation. Furthermore, the addition of a third element M is also examined as Nd${\;}_{0.67}$Fe${\;}_{0.33-y}M_{y}$ alloys, where M stands for Al, Co, Cu, and Ga. Randomness in the atomic configuration of antisites, vacancies, and third-element atoms at the Fe sites are considered by the SQS method [45]. The formation energy $E_{form}$ for $Nd_{x}Fe_{1-x-y}M_{y}$ is calculated as

$$E_{form}=E_{Nd_{x}Fe_{1-x-y}M_{y}}-\left[x\mu_{Nd}+\left(1-x-y\right)\mu_{Fe}+y\mu_{M}\right],$$

where $E_{Nd_{x}Fe_{1-x-y}M_{y}}$ is the total energy of the $Nd_{x}Fe_{1-x-y}M_{y}$ alloy per atom and $\mu_{Nd},\mu_{Fe}$, and $\mu_{M}$ are the chemical potentials of double hexagonal close-packed (dhcp) Nd, bcc Fe, and M with the most stable crystal structures, respectively.

**Figure 7.**
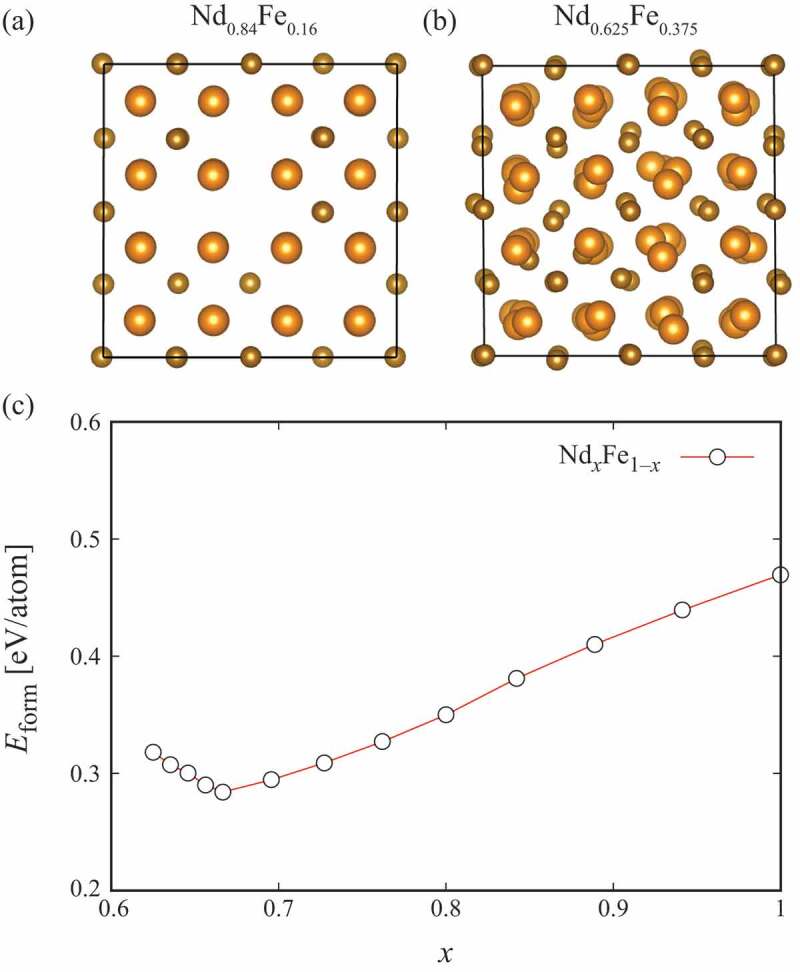
The optimized crystal structures of the fluorite Nd-Fe alloys for (a) Nd${\;}_{0.8}$Fe${\;}_{0.2}$ and (b) Nd${\;}_{0.625}$Fe${\;}_{0.375}$. (c) The formation energies $E_{form}$ of the fluorite Nd-Fe alloys. The figure is taken from [72]. Copyright (2020) The Authors

Figure 7(a) shows the Nd${\;}_{x}$Fe${\;}_{1-x}$ crystal structure with the composition increased from x=0.67. It is found that the Fe-vacancy formation is more stable than the substitution of Fe with Nd. The crystal structure is hardly distorted by increasing x. In contrast, the Nd-site substitution with Fe is seen rather than the Nd-vacancy formation for decreased x from x=0.67. Structural distortion is found by decreasing x as shown in Figure 7(b). Figure 7(c) shows the formation energy $E_{form}$ for the crystalline Nd${\;}_{x}$Fe${\;}_{1-x}$ alloys. With its minimum at x=0.67, $E_{form}$ gradually increases as x varies from the stoichiometric composition. It should be noted that another structure should become more stable for x≈1, because the structure becomes simple cubic for pure Nd in this case. From the structure optimization, it is concluded that the structure becomes completely amorphous for x<0.6, which is consistent with experiments observing the crystalline intergranular phase mainly for 0.6<x<0.7 [42]. Positive formation energies for binary Nd${\;}_{x}$Fe${\;}_{1-x}$ means that the configurational entropy at finite temperatures is indispensable to promote the alloy formation avoiding the decomposition into dhcp Nd and bcc Fe. As for magnetism, Nd${\;}_{x}$Fe${\;}_{1-x}$ exhibits magnetic moments of $1.9\mu_{B}$ by Fe 3d and $-0.53\mu_{B}$ by Nd 5d that are practically independent of x.

**Figure 8.**
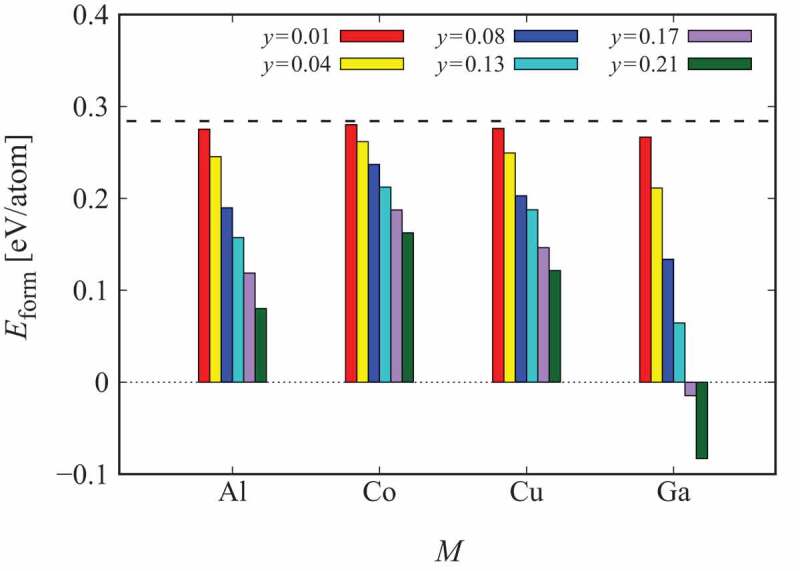
Formation energies of fluorite $Nd_{0.67}Fe_{0.33-y}M_{y}$ alloys. The dashed line indicates the formation energy of the binary Nd${\;}_{0.67}$Fe${\;}_{0.33}$ alloy with the fluorite structure. For y=0.17, the composition should be read as $Nd_{2/3}Fe_{1/6}M_{1/6}$. The figure is taken from [72]. Copyright (2020) The Authors

The third-element effect on the stability of the crystalline Nd-Fe structure is discussed. Experimentally, the addition of Cu and Ga to Nd-Fe-B permanent magnets has been reported with the improvement of the coercivity [69–71], where Cu particularly decreases the melting point of Nd making the wettability of Nd-Fe alloys better. Figure 8 illustrates the formation energies of the ternary Nd${\;}_{0.67}$Fe${\;}_{0.33-y}M_{y}$ systems, where M is Al, Co, Cu, or Ga. It is clear that the third elements decrease the formation energy. Among the four elements, Ga is the most efficient to stabilize the crystal structure [72]. This result is consistent with experiments where a crystalline intergranular phase has been found in Ga-added Nd-Fe-B magnets [43].

**Figure 9.**
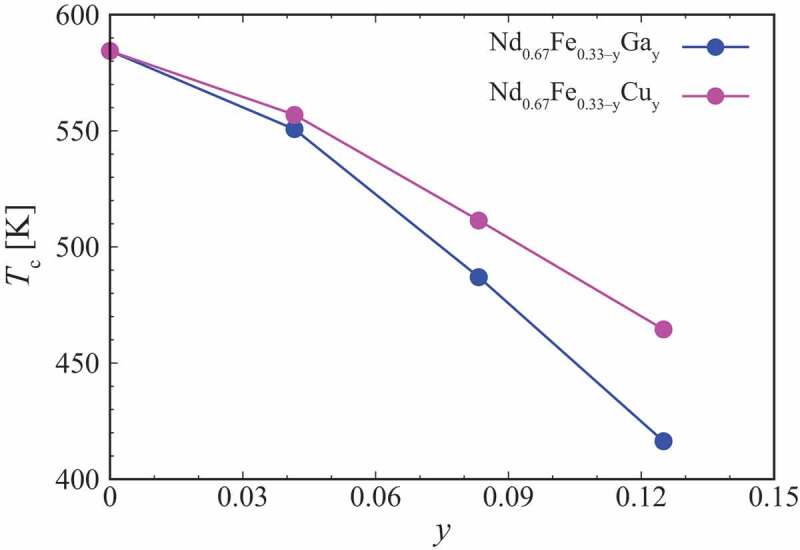
Curie temperature $T_{C}$ for fluorite Nd-Fe-M alloys as a function of the composition parameter y. The figure is taken by permission from [74]. Copyright (2020) The Japan Society of Applied Physics

Finite-temperature magnetism of crystalline Nd-Fe alloys with the addition of Cu or Ga is discussed from first principles. Exchange coupling constant $J_{ij}$ is calculated within the KKR-CPA scheme by the Akai-KKR code [46], where the magnetic ground state is used as a reference. The Curie temperature is estimated qualitatively by the mean-field approximation. The substitution of Fe with Ga or Cu at the fluorite 4a sites is treated by the CPA for convenience. Figure 9 shows the Curie temperature $T_{C}$ of Nd${\;}_{0.67}$Fe${\;}_{0.33-y}M_{y}$ alloys. The Curie temperature is quantitatively expected to be lower than the values obtained in this study, because the overestimation of $T_{C}$ within the same theoretical scheme has been found for Nd${\;}_{2}$Fe${\;}_{14}$B [73]. It is clear from the figure that the addition of Ga and Cu is effective in decreasing $T_{C}$ that is suitable for an intergranular phase. In addition, Ga is more effective than Cu to make Nd-Fe alloys paramagnetic at room temperature. The addition of Ga weakens the exchange interaction among Nd−Fe as well as Fe−Fe pairs, which results in the decrease in $T_{C}$ [74].

## 5. Summary

Microscopic insights into microstructures can be obtained by first-principles calculations for surfaces of the main phase and interfaces between the main phase and the intergranular phase. Magnetic coupling between main-phase grains as well as local magnetic properties at the interface are of particular interest. Such local magnetic properties will be used in spin models to investigate magnetization reversal. Microstructure interfaces in commercial magnets are in strongly non-equilibrium states having disorders. Thus, large-scale computations by supercomputers such as Fugaku are expected to be useful.

Theoretical description of finite-temperature magnetism is still insufficient. By considering the indirect effects of magnetism-dependent phonons on magnetism, the Curie temperature can change by more than 500 K, which had been completely overlooked. In addition to understanding of microstructure effects, better descriptions of finite-temperature magnetism and applications to magnetic compounds involved in permanent magnets should stimulate further progress in the clarification of coercivity mechanism on the basis of electron theory.
